# Editorial: Roles of the Circadian Rhythms in Metabolic Disease and Health

**DOI:** 10.3390/metabo14110621

**Published:** 2024-11-14

**Authors:** Letizia Galasso, Lucia Castelli, Eleonora Bruno

**Affiliations:** 1 Department of Biomedical Sciences for Health, University of Milan, 20133 Milan, Italy; letizia.galasso@unimi.it; 2 Nutrition Research and Metabolomics Unit, Department of Experimental Oncology, Fondazione IRCCS Istituto Nazionale dei Tumori di Milano, 20133 Milan, Italy; eleonora.bruno@istitutotumori.mi.it

Chronobiology is the field of study focused on understanding the temporal patterns of biological functions, specifically examining the regular cycles or oscillations in these processes [1]. Since almost all biological functions exhibit periodic variations, this area of research covers a wide range of topics, from fundamental biological studies to medical applications. Human biology is inherently time-bound, with rhythms observable at various levels of biological organization. Among these, circadian rhythms are the most thoroughly investigated [2]. The word “circadian” is derived from the Latin terms circa, meaning “about”, and diem, meaning “day”, referring to processes that cycle approximately every 24 h (with a range of 20–28 h) [1]. Circadian rhythms guide daily behaviors and evolve throughout the lifespan [3,4], potentially shifting in response to both healthy and pathological conditions [5,6,7,8,9]. These rhythms are regulated by a core set of circadian clock genes that function in a feedback loop, controlling the timing and oscillations of biological cycles [10,11,12]. However, circadian rhythms, such as the sleep–wake cycle, are also influenced by external stimuli [13,14,15,16]. Lifestyle and environmental factors of modern societies can induce disruptions in the circadian system and thereby adversely affect health. Recent research has explored how disruptions in circadian rhythms and the sleep–wake cycle are linked to various metabolic and health-related conditions, including hypertension, obesity, dyslipidemia, diabetes, neurodegenerative diseases, and even cancer [7,13,17,18,19,20,21,22,23]. The causes of circadian rhythm alterations are numerous and not yet fully understood. Among these, modern lifestyles, including shift work, excessive exposure to artificial light, reduced physical activity, and poor dietary habits, can adversely affect the human circadian system, leading to disruptions in biological rhythms [24,25,26,27,28]. Growing evidence demonstrates a complex reciprocal relationship between metabolism and the circadian system, wherein perturbations in one affect the other. Diet is one of the key synchronizers of our internal clock mechanisms; thus, abnormal feeding times can lead to a misalignment between environmental oscillators and the central pacemaker, resulting in adverse health outcomes. In particular, alterations in the cycle between periods of eating and fasting have been associated with a predisposition to nutrition-related diseases, including obesity, type 2 diabetes (T2DM), and cardiovascular disease (CVD) [29,30,31].

The current Special Issue, “Roles of the Circadian Rhythms in Metabolic Disease and Health”, features five original research articles and one systematic review, showcasing recent advances on the roles of circadian rhythms and their disruptions in (i) metabolic and glucose homeostasis; (ii) blood levels of melatonin and ornithine supplementation; (iii) diet and its impact on circadian clocks.

Among the contributions, several studies provide in-depth examinations of specific mechanisms by which circadian disruptions influence metabolic outcomes.

The study by Her et al. (2024) (contribution 1) focused on the effects of circadian disruption on mice’s metabolism. Initially, all mice were kept under a normal day cycle of 12 h/12 h light/dark. Before mating, part of the group was shifted to the short-day group under a short-day (SD) cycle of 8 h/8 h light/dark. Thus, the offsprings of the control group (Ctrl) were maintained under 12 h/12 h light/dark conditions, whereas those of the short-day group lived under 8 h/8 h light/dark conditions until the end of the study. All Ctrl and SD offspring were fed a normal chow diet (NCD) for the first 7–10 weeks after weaning and later switched to a high-fat diet (HFD). Disruption of circadian rhythms throughout life due to environmental light exposure results in metabolic impairments in adult mice. Given that alterations to circadian rhythms during prenatal and early life periods affect metabolism in a sexually dimorphic manner, the authors showed that exposure to an 8 h light/8 h dark cycle worsens glucose tolerance and promotes insulin resistance in female mice. On a molecular level, glucose intolerance appears to be driven, in part, by insulin resistance and disrupted insulin signaling in both skeletal muscle and liver tissues. When combined with HFD, both male and female mice exhibit heightened vulnerability to obesity-related challenges and metabolic dysfunctions. Collectively, these findings suggest that environmental disruptions to circadian rhythms throughout life may increase the risk of developing obesity and T2DM in adulthood.

Glucose homeostasis and alteration was also investigated in the study by Yong et al. (2023) (contribution 2), which recruited 36 participants with a 2 × 2 acute randomized crossover study and assessed the interactions of low or high glycemic index (GI) with the timing of food intake (breakfast or dinner) on glucose homeostasis. Blood samples were subsequently drawn at regular intervals over a period of 3 h, at 30, 60, 120, and 180 min after breakfast (09:00) and dinner (18:30). The findings suggest that, overall, individuals’ metabolic states tend to show a higher risk for cardiometabolic diseases in the evening compared to the morning. This observation raises the possibility that consuming a larger proportion of nutrient-dense foods with low GI in the evening could help reduce this risk. The authors noted that 29 out of 234 (approximately 12%) of the tested metabolites showed significant differences in the postprandial area under the curve (AUC) between the breakfast and dinner groups. The differences between breakfast and dinner were much more pronounced than those observed between meals with low and high GI. In fact, only 5 out of 234 (2%) metabolites demonstrated significant differences between the high GI and low GI groups, suggesting that the GI itself has a relatively minor effect on the metabolites measured. These metabolic changes may provide insights into potential molecular signatures or pathways that link meal timing and GI to metabolic responses and the risk of cardiometabolic diseases.

Other risks for cardiometabolic diseases have been highlighted in the study by Bizzarri et al. (2022) (contribution 3), which used data from the “Lifelines Cohort Study”, selecting 2020 participants, divided into 1010 shift workers and 1010 non-shift workers (938 women: *n* = 469 non-shift workers and *n* = 469 night-shift workers; 1082 men: *n* = 541 non-shift workers and *n* = 541 night-shift workers). Participants underwent blood sampling to measure 250 metabolomic biomarkers. Both genders showed differences in body mass index (BMI) related to night work, but metabolic changes were only observed in male night-shift workers. Specifically, men showed increased levels of glycoprotein acetyls (GlycA), triglycerides, and fatty acids. These conclusions are relevant for the health status, since it has been hypothesized that GlycA, a marker of chronic inflammation, may be linked to higher risks of obesity, T2DM, and CVD, as seen in several cohort studies, as well as an increased risk of severe infections.

Regarding the role of melatonin and ornithine in circadian rhythms and health, the study by Milanowski et al. (2023) (contribution 4) involved 64 participants, divided into three groups: 20 individuals in the early stages of Parkinson’s disease (PD) without dyskinesia, 24 individuals in advanced stages of PD with dyskinesia, and 20 healthy controls. All participants in the PD groups were undergoing pharmacological treatment, combining benserazide with levodopa. Blood samples were collected from all participants to measure biochemical parameters, such as melatonin, leptin, adiponectin, and resistin levels. The results showed that individuals in the early stages of PD had the lowest levels of melatonin when compared to both the control group and those with advanced PD, suggesting that impaired melatonin secretion may contribute to the onset of PD, and that early PD symptoms, such as sleep disturbances, could be associated with its deficiency. Melatonin supplementation might be beneficial in preventing or alleviating early-stage PD symptoms. Interestingly, in Milanowski et al.’s (2023) study, participants with advanced PD exhibited higher melatonin levels than those in the early stages or the healthy controls. This finding suggests that melatonin concentration may increase as the disease progresses. The treatments with levodopa, a dopamine precursor, could be linked to higher melatonin levels in PD patients with dyskinesia. In addition to melatonin, abnormalities in adipokine levels were observed, particularly in leptin and resistin. Low leptin levels seem to be associated with both the onset and progression of PD. Notably, resistin levels were elevated only in those with advanced PD, suggesting that resistin may play a role in the progression of the disease towards dyskinesia. Although changes in adiponectin levels were not statistically significant, a positive correlation between its concentration and disease severity was observed.

With regard to the role of supplementation in improving sleep–wake cycle and health, Takakura et al. (2023) (contribution 5) explored the potential role of L-ornithine supplementation in promoting sleep-like behavior in mouse pups. L-ornithine is a non-proteinogenic amino acid, which has been reported to have both sedative and hypnotic effects in neonatal chicks, as well as an anxiolytic-like effect in mice. Additionally, L-ornithine promotes NREM sleep in mice and improves sleep quality in humans. In this article, the authors conducted three experiments to explore the effects of dietary L-ornithine. They investigated maternal plasma and milk L-ornithine levels in mice, the time-course changes in plasma, mammary gland, and milk L-ornithine levels following a single dose of L-ornithine administration, and finally, sleep behavior changes alongside modifications in polyamine levels in milk. The results revealed that while maternal ingestion of L-ornithine increased L-ornithine levels in breast milk, it was not sufficient to promote sleep in newborns. Given the challenge of raising L-ornithine levels in breast milk to a sleep-inducing threshold, L-ornithine-enriched formula may partially improve infant sleep and holds potential for preventing sleep problems, such as nighttime crying, in infants.

Further, considering the critical role of food in synchronizing peripheral clocks, alongside metabolism, which influences both central and peripheral clocks, and that disruption of circadian system can contribute to cardiometabolic diseases, Paula et al. (2024) (contribution 6) examined the effects of diet, meal timing, and dietary restrictions on the heart’s circadian clock in mice, highlighting how these factors impact cardiovascular health. Their review emphasizes that meal timing significantly affects circadian rhythms in peripheral organs like the heart and liver. Restricted feeding (RF), where food is available for limited hours without reducing calorie intake, influences clock gene expression, particularly in the heart. Feeding during the light phase can cause desynchronization between metabolically active tissues, like the liver and heart, and alter metabolic pathways. Some studies also found that feeding during the dark phase increased calorie intake and affected fatty acid oxidation. RF affected clock gene expression in both wild-type and PPARα-deficient mice, with specific genes like Bmal1 showing altered expression patterns, leading to changes in heart function and energy reserves. Diet composition, including fats, carbohydrates, proteins, and micronutrients, also impacts the circadian clock. High-fat diets (HFD) disrupted circadian rhythms, altered cardiac metabolism, and induced changes linked to CVD. HFD-induced obesity affected lipid metabolism and cardiac remodeling, but meal timing (restricted to the dark phase) reversed some of these effects. Caloric restriction (CR), reducing intake by 30%, had protective effects on ischemic hearts by altering gene expression related to antioxidants and circadian rhythms, suggesting a protective role of heart clock genes during ischemic events. The ketogenic diet (KD) similarly influenced circadian rhythms through changes in cellular energy status, while diets rich in branched-chain amino acids (BCAAs), fiber, and biotin also impacted heart health and circadian regulation. Overall, the review highlights that both diet composition and feeding schedules significantly influence peripheral clocks, especially in the heart, affecting cardiac metabolism and the expression of clock genes.

In conclusion, this Special Issue underscores the crucial role of circadian rhythms in maintaining metabolic homeostasis and preventing diseases. Alterations caused by external factors like night work, diet, and artificial light exposure can negatively impact health, suggesting that targeted interventions to realign circadian rhythms may help mitigate the risk of metabolic and neurodegenerative diseases.
